# Neutralization activity of IgG antibody in COVID‑19‑convalescent plasma against SARS-CoV-2 variants

**DOI:** 10.1038/s41598-023-28591-3

**Published:** 2023-01-23

**Authors:** Kiyoto Tsuchiya, Kenji Maeda, Kouki Matsuda, Yuki Takamatsu, Noriko Kinoshita, Satoshi Kutsuna, Tsunefusa Hayashida, Hiroyuki Gatanaga, Norio Ohmagari, Shinichi Oka, Hiroaki Mitsuya

**Affiliations:** 1 AIDS Clinical Center, National Center for Global Health and Medicine, Tokyo, Japan; 2 Department of Refractory Viral Infections, National Center for Global Health and Medicine Research Institute, Tokyo, Japan; 3 Disease Control and Prevention Center, National Center for Global Health and Medicine, Tokyo, Japan; 4 Joint Research Center for Human Retrovirus Infection, Kumamoto University, Kumamoto, Japan; 5 Experimental Retrovirology Section, HIV and AIDS Malignancy Branch, National Cancer Institute, National Institutes of Health, Bethesda, MD USA; 6 Department of Clinical Sciences, Kumamoto University Hospital, Kumamoto, Japan

**Keywords:** SARS-CoV-2, Viral infection

## Abstract

Coronavirus disease 2019 (COVID-19) is caused by severe acute respiratory syndrome coronavirus 2 (SARS-CoV-2). We evaluated the anti-SARS-CoV-2 antibody levels, anti-spike (S)-immunoglobulin G (IgG) and anti-nucleocapsid (N)-IgG, and the neutralization activity of IgG antibody in COVID‑19‑convalescent plasma against variants of SARS-CoV-2, alpha, beta, gamma, delta, kappa, omicron and R.1 strains. The study included 30 patients with clinically diagnosed COVID-19. The anti-S-IgG and anti-N-IgG levels ranged from 30.0 to 555.1 and from 10.1 to 752.6, respectively. The neutralization activity (50% inhibition concentration: IC_50_) for the wild-type Wuhan strain ranged from < 6.3 to 81.5 µg/ml. IgG antibodies were > 100 µg/ml in 18 of 30 (60%) subjects infected with the beta variant. The IC_50_ values for wild-type and beta variants correlated inversely with anti-S-IgG levels (*p* < 0.05), but no such correlation was noted with anti-N-IgG. IgG antibodies prevented infectivity and cytopathic effects of six different variants of concern in the cell-based assays of wild-type, alpha, gamma, delta, kappa and R.1 strains, but not that of the beta and omicron strains. IgG is considered the main neutralizing activity in the blood, although other factors may be important in other body tissues.

## Introduction

Severe acute respiratory syndrome coronavirus 2 (SARS-CoV-2) causes coronavirus disease 2019 (COVID-19), which emerged in China at the end of 2019^1–5^ and spread rapidly worldwide. Genetic variants of SARS-CoV-2 have emerged and are currently circulating around the world. They define three classes of SARS-CoV-2 variants^6^: the B.1.1.7 (alpha), B.1.351 (beta), P.1 (gamma), B.1.617.2 (delta) and B.1.1.529 (omicron) variants, which are classified as variants of concern (VOC), while B.1.617.1 (kappa) and R.1 variants are classified as variants of interest (VOI). Variant mutations in these viruses are associated with changes in the activity of receptor binding and reduced neutralization by antibodies^7–9^.

Patients infected with COVID-19 produce various antibodies, including immunoglobulin M (IgM), immunoglobulin G (IgG) and immunoglobulin A (IgA)^10–12^. Especially, IgG antibodies against the spike (S) protein containing the anti-receptor binding domain (RBD) and nucleocapsid (N) protein of SARS-CoV-2 prevent the acquisition of viral infection^13^. The amounts of anti-S-IgG and anti-N-IgG antibodies produced after natural SARS-CoV-2 infection in unvaccinated individuals are more than 10 times those in negative samples upon admission and more than 100 times those at convalescence^14^.

**Figure 1:**
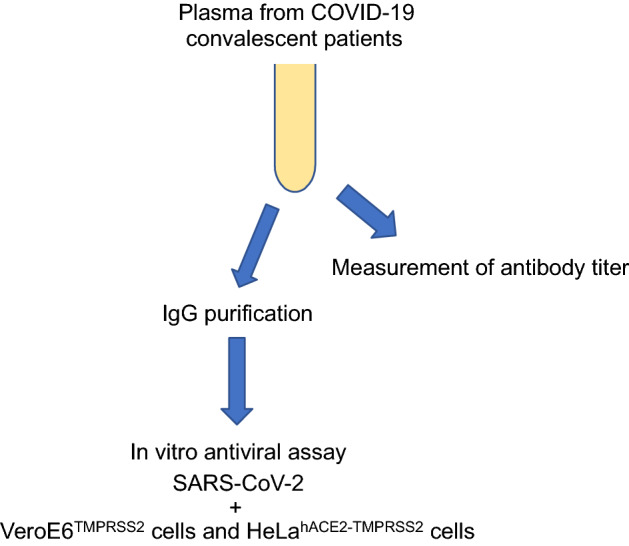
Schematic diagram of the IgG antibody neutralization assay of COVID-19 convalescent plasma.

The purpose of this study was to determine the neutralization activities of IgG antibodies from COVID‑19‑convalescent plasma against variants of SARS-CoV-2, alpha, beta, gamma, delta, kappa, omicron and R.1 strains (Fig. 1).

## Results

### Anti-SARS-CoV-2 antibody titers

**Figure 2:**
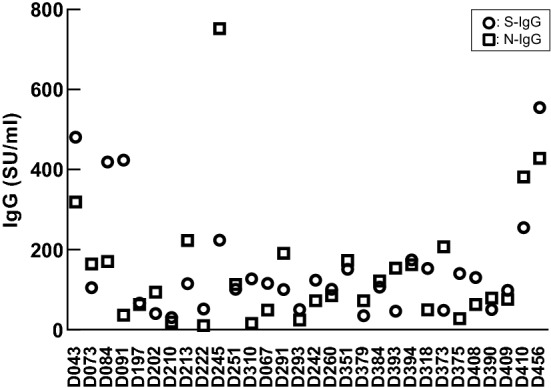
Anti-SARS-CoV-2 antibody titers in individual subjects.

Figure 2 shows the levels of anti-S-IgG and anti-N-IgG measured in the 30 study patients. The anti-S-IgG and -N-IgG levels ranged from 30.0 to 555.1, and from 10.1 to 752.6, respectively.

### Antiviral activity of IgG antibody in convalescent plasma samples

**Figure 3:**
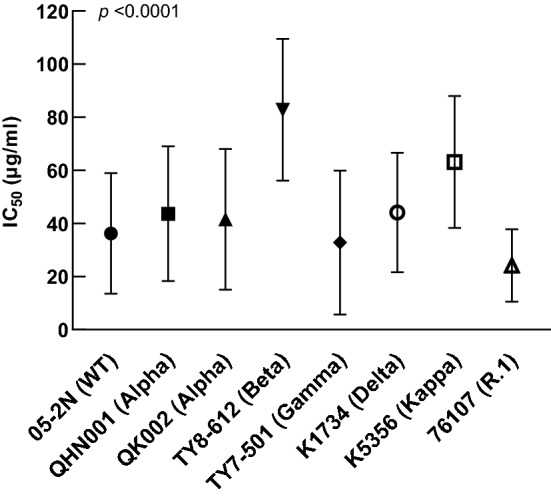
Antiviral activity of IgG antibodies in convalescent plasma against wild-type and each variant. Data are mean ± standard deviation.

We evaluated the antiviral activity of IgG antibody in convalescent plasma against the wild-type (WT) and variants, alpha, beta, gamma, delta, kappa and R.1 strains. Table 1 shows the IC_50_ of IgG antibodies against WT and variant strains in VeroE6^TMPRSS2^ cells. The IC_50_ varied from < 6.3 to 81.5 µg/ml for the WT, alpha, gamma, delta, kappa and R.1 variants. On the other hand, the IC_50_ of IgG antibodies against the beta variant was > 100 µg/ml in 18 of 30 (60%) subjects. Figure 3 shows the mean IC_50_ of all the antibodies. The mean ± SD IC_50_ values for the alpha (43.68 ± 25.36 µg/ml in QHN001, 41.54 ± 26.52 µg/ml in QK002), gamma (32.86 ± 27.14 µg/ml), delta (44.14 ± 22.52 µg/ml) and R.1 (24.18 ± 13.62 µg/ml) variants were similar to that of WT (36.25 ± 22.72 µg/ml), but the values for the beta (82.86 ± 26.68 µg/ml) and kappa (63.19 ± 24.82 µg/ml) variants in VeroE6^TMPRSS2^ cells were clearly higher than WT. The mean IC_50_ of beta was significantly higher than those of WT, alpha, gamma, delta, and R.1 (*p* < 0.0001, Kruskal–Wallis test). The post-hoc Dunn’s multiple comparisons test showed statistically significant difference in mean IC_50_ between beta and WT (*p* < 0.0001), alpha (*p* < 0.0001), gamma (*p* < 0.0001), delta (*p* < 0.01), and R.1 (*p* < 0.0001), and also in the mean IC_50_ of kappa and WT (*p* < 0.01), gamma (*p* < 0.001), and R.1 (*p* < 0.0001).

**Table 1:** Convalescent plasma antiviral activity of IgGs in VeroE6^TMPRSS2^.

IgG	05-2N	QHN001	QK002	TY8-612	TY7-501	K1734	K5356	76,107
	B	B.1.1.7	B.1.1.7	B.1.351	P.1	B.1.617.2	B.1.617.1	R.1
	WT	Alpha	Alpha	Beta	Gamma	Delta	Kappa	–
D043	11.9	20.3	18.1	30.7	10.7	39.7	31.0	7.0
D073	63.9	53.2	78.9	> 100	58.3	> 100	> 100	42.2
D084	28.8	29.4	23.7	73.6	14.6	59.3	74.0	14.3
D091	54.5	> 100	> 100	> 100	39.0	> 100	> 100	51.8
D197	16.2	34.6	27.9	> 100	64.1	41.2	71.1	39.1
D202	74.7	> 100	66.8	> 100	> 100	47.8	89.8	53.9
D210	61.3	65.5	49.3	> 100	54.9	55.8	93.6	51.6
D213	29.5	26.5	20.2	> 100	49.9	35.9	51.5	23.2
D222	69.5	54.9	26.3	> 100	22.0	> 100	> 100	29.5
D245	22.9	18.5	10.6	> 100	8.0	42.4	60.2	28.1
D251	30.3	26.2	16.1	> 100	6.9	29.1	52.6	23.0
D310	28.4	17.2	15.0	59.2	9.3	36.7	61.7	28.9
D067	81.5	64.2	> 100	> 100	85.2	42.0	47.2	25.6
D291	36.0	28.6	48.7	> 100	59.8	42.8	40.0	19.3
D293	63.8	40.2	70.9	> 100	87.8	59.7	> 100	29.8
D242	44.7	64.4	61.3	> 100	21.7	58.6	56.1	24.6
D260	19.2	13.6	7.5	18.6	< 6.3	52.2	55.4	< 6.3
D351	40.2	32.2	22.1	> 100	17.0	49.7	84.7	22.0
D379	78.3	> 100	62.5	67.8	53.0	29.2	34.2	14.4
D384	20.0	36.3	27.4	85.9	12.7	30.3	47.8	20.3
D393	43.6	64.1	54.9	> 100	23.6	26.3	57.9	17.3
D394	46.7	47.2	63.7	> 100	46.3	42.6	> 100	41.2
D318	10.4	27.7	28.0	75.9	9.5	26.1	40.6	12.0
D373	19.9	51.9	66.0	> 100	20.0	18.0	34.5	15.5
D375	17.1	33.2	39.0	78.5	18.1	25.3	> 100	21.3
D408	19.0	58.6	56.7	90.8	22.1	26.8	38.5	13.9
D390	27.6	53.2	41.6	> 100	41.0	27.9	42.9	25.8
D409	7.6	13.6	10.1	17.1	< 6.3	42.7	63.7	8.1
D410	13.6	22.1	16.0	52.1	10.2	20.3	36.5	9.1
D456	< 6.3	13.0	16.9	35.7	7.4	15.9	30.3	< 6.3
mAb1414	0.72	> 10	NT	> 10	> 10	> 10	> 10	5.12
mAb2414	5.78	> 10	NT	> 10	3.46	> 10	> 10	> 10
mAb40591	3.27	5.46	NT	> 10	0.88	2.62	> 10	1.35
pAbA19215	< 0.63	< 0.63	NT	> 10	> 10	< 0.63	5.56	< 0.63

Data are IC_50_ (µg/ml) values.

We also evaluated the activities of anti-SARS-CoV-2 antibodies. All the four tested antibodies showed potent activities against the WT strain, however, the activities were markedly lower in most variant strains, especially mAb1414 and 2414 nullified activity, in almost all tested variants (Table 1). Table 2 shows the IC_50_ of IgG antibodies^15^ against WT and variants strains in HeLa^hACE2-TMPRSS2^ cells. Note the higher IC_50_ values for the beta and omicron variants relative to the WT.

**Table 2:** Convalescent plasma antiviral activity of IgGs in HeLa^hACE2-TMPRSS2^.

Cell	IgG	05-2N	TY8-612	K1734	929-1N
		B	B.1.351	B.1.617.2	B.1.1.529
		WT	Beta	Delta	Omicron
VeroE6 TMPRSS2	D043	11.9	30.7	39.7	n.d.
	D073	63.9	> 100	> 100	n.d.
	D084	28.8	73.6	59.3	n.d.
	D091	54.5	> 100	> 100	n.d.
HeLa hACE2-TMPRSS2	D043	7.9	17.5	9.4	17.9
	D073	40.7	> 100	53.7	> 100
	D084	8.9	65.3	22.8	> 100
	D091	30.0	93.1	72.4	59.0

Data are IC_50_ (µg/ml) values.

### Correlation between neutralizing activity against WT/beta variant and antibody titers

**Figure 4:**
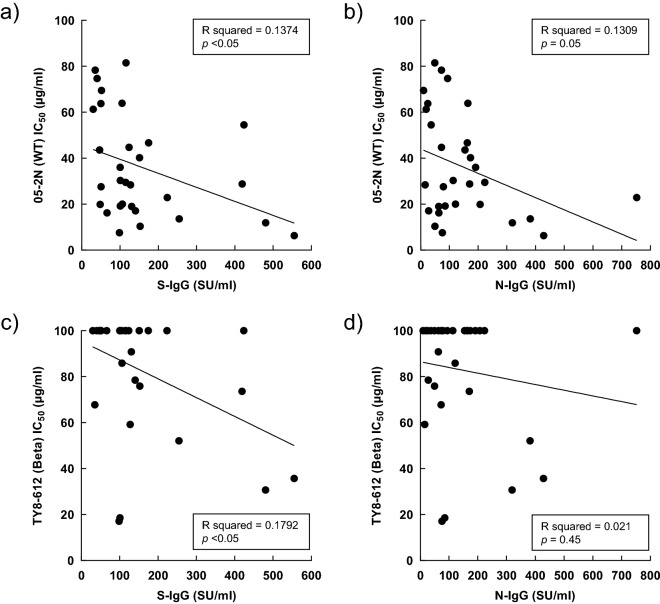
Correlation between neutralizing activity against antibody titers and wild-type and beta variant. (**a**) Wild-type vs. anti-S-IgG levels. (**b**) Beta variant vs. anti-S-IgG levels. (**c**) Wild-type vs. anti-N-IgG levels. (**d**) Beta variant vs. anti-N-IgG levels.

Finally, we compared the correlation between neutralizing activities against WT, beta variant and antibody titers. The IC_50_ values in WT and beta correlated significantly with anti-S-IgG levels (*p* < 0.05), but not with anti-N-IgG levels (Fig. 4a–d).

## Discussion

Genetic variants of SARS-CoV-2, alpha, beta, gamma, delta, kappa, omicron and R.1 strains, have infected millions of people around the world^6^. We evaluated the antiviral activities of IgG antibodies against those variants obtained from convalescent plasma samples. The IgG antibodies prevented the infectivity and cytopathic effects of three different VOCs and two VOIs in the cell-based assays using various infectious variants, WT, alpha, gamma, delta, kappa, and R.1 strains, but not those of the beta and omicron strains. These results are somewhat similar to those reported on the neutralizing activities found in BNT162b2-vaccinated individuals, which demonstrated potent activities against the alpha, delta, and kappa variants in serum samples from responders, compared with relatively moderate activity against the beta strain^16,17^. As described in the Methods section, the tested plasma samples were obtained from patients who were infected with COVID-19 between June 2020 and March 2021, and thus, it is considered that most IgGs were from convalescent plasmas of non-VOCs (Nextstrain clade 20B). Therefore, it is reasonable that the results of vaccines (prepared based on the sequence of WT) and that of the convalescent plasma samples tended to be the same^7,16–18^. We evaluated the correlation between the levels of the antibodies and neutralization activities of IgG antibodies. The higher levels of anti-S-IgG tended to suppress acquisition of viral infection. Then, the anti-S-IgG levels correlated inversely with the IC_50_ values for the WT and beta variant. In other words, anti-S-IgG antibody prevented the infectivity and cytopathic effects in SARS-CoV-2 infection. These results indicate that anti-S-IgG antibody acts directly against the RBD of SARS-CoV-2, preventing viral entry into the cells. Similar results were reported on the correlation between anti-S-IgG levels and neutralizing activity of COVID‑19‑convalescent plasma^19,20^. In contrast, none of the anti-N-IgG levels correlated inversely with the IC_50_ values for the WT and beta variant. These results suggest that anti-N-IgG antibody is unlikely to prevent SARS-CoV-2 infection in cells. Nevertheless, one previous study reported that anti-N-IgG antibody correlated with the neutralizing efficacy of a SARS-CoV-2 pseudovirus in all randomly selected COVID‑19‑convalescent plasma units^21^. Further analysis of the correlation between anti-N-IgG antibody and neutralizing activity is warranted.

Our study has certain limitations. First, we did not determine the variants of SARS-CoV-2 from COVID‑19‑convalescent patients. However, WT and the initial alpha strains were consistent with the variants that had spread in Japan during the study period^22^. Second, the small sample size is a limitation of this study as it reduces the generalizability of the findings to a larger population. Further research with a larger sample size is needed to confirm these results. In this study, the IgG was purified and used to evaluate the activity of COVID‑19‑convalescent plasma, and the results correlated with S-IgG in the blood. IgG is considered to be the main neutralizing activity in the blood, although other factors may be important in neutralizing the activity in the lungs, mucous membranes, and other tissues.

## Materials and methods

### Patients

The study subjects were 30 patients (22 men and 8 women, median age 54; range: 25–69 years) diagnosed clinically with COVID-19 for the first time after visiting the National Center for Global Health and Medicine (NCGM), Tokyo, Japan, between June 2020 and March 2021. Twenty five of the 30 (83.3%) patients developed co-existing severe pneumonia, and of these, 5 required positive pressure ventilation, and 3 of the latter also required treatment with extracorporeal membrane oxygenation. Plasma test samples were obtained between 33 and 316 days (median 96 days) after the onset of clinical features of COVID-19 infection. The Human Ethics Committee of the NCGM approved the study (#NCGM-G-003472–02) and each patient provided a written informed consent. The study also conformed to the principles of the Declaration of Helsinki.

### Measurement of anti-SARS-CoV-2 antibody titers

Samples from each participant were analyzed for the levels of two anti-SARS-CoV-2 antibodies (anti-S-IgG and anti-N-IgG) using the chemiluminescence enzyme immunoassay (CLEIA) platforms (HISCL) purchased from Sysmex Co. (Kobe, Japan) as reported previously^14^.

### Cells, viruses, antibodies and isolation of IgG fractions from COVID‑19‑convalescent patients

Vero-E6^TMPRSS2^ cells^23^ and HeLa^hACE2-TMPRSS2^ cells^24^ were obtained from Japanese Collection of Research Bioresources (JCRB) Cell Bank (Osaka, Japan). Each cell type was maintained in D-MEM supplemented with 10% FCS, 100 μg/ml of penicillin, 100 μg/ml of streptomycin, and 1 mg/mL of G418. PANGO lineage B, wild type (WT) Wuhan strain [SARS-CoV-2 NCGM-05-2N (SARS-CoV-2^05-2N^)] and B.1.1.529 (omicron) variants [hCoV-19/Japan/IC-2279/2021 SARS-CoV-2 NCGM-929-1N (SARS-CoV-2^929-1N^)] were isolated from nasopharyngeal swabs of a patient with COVID-19, who was admitted to the NCGM^7,13,16,17^. Seven clinically isolated SARS-CoV-2 mutant strains were used in the present study: two B.1.1.7 (alpha) variants [hCoV-19/Japan/QHN001/2020 (SARS-CoV-2^QHN001^, GISAID accession ID; EPI_ISL_804007) and hCoV-19/Japan/QK002/2020 (SARS-CoV-2^QK002^, GISAID Accession ID; EPI_ISL_768526)] and a B.1.351 (beta) variant [hCoV-19/Japan/TY8-612-P0/2021 (SARS-CoV-2^TY8-612^, GISAID accession ID; EPI_ISL_1123289)] and a P.1 (gamma) variant [hCoV-19/Japan/TY7-501/2021 (SARS-CoV-2^TY7-501^, GISAID Accession ID; EPI_ISL_833366)] were obtained from the National Institute of Infectious Diseases, Tokyo. The B.1.617.2 (delta) variant [hCoV-19/Japan/TKYK01734/2021 (SARS-CoV-2^1734^, GISAID Accession ID; EPI_ISL_2080609)], B.1.617.1 (kappa) variant [TKYTK5356_2021 (SARS-CoV-2^5356^, DDBJ Accession ID; LC633761)] and R.1 variant [hCoV-19/Japan/TKY76107/2021 (SARS-CoV-2^76107^, GISAID Accession ID; EPI_ISL_1041946)] were provided by Tokyo Metropolitan Institute of Public Health, Tokyo. Each variant was confirmed to contain each variant of concern-specific amino acid substitutions before the assays conducted in the present study (vide infra). The mAb1414, mAb2414 and mAb40591, anti-SARS-CoV-2 monoclonal antibodies, were purchased from Active Motif (Carlsbad, CA) and Sino Biological (Beijing, China), respectively. The pAbA19215, anti-SARS-CoV-2 polyclonal antibody was purchased from ABclonal (Woburn, MA). Plasma or serum samples were collected from patients, and IgG fractions were purified using a spin column-based antibody purification kit (Cosmo Bio, Tokyo) according to the instructions provided by the manufacturer. Briefly, serum or plasma was collected, heat-inactivated for 30 min at 56 °C, and spin columns were centrifuged at 3500 rpm for 5 min. The IgG fractions in the supernatants were eluted and collected.

### Antiviral assays

The neutralizing activities of IgG fractions from COVID‑19‑convalescent plasma were determined by quantifying the IgG antibody suppression of the cytopathic effect (CPE) of each SARS-CoV-2 strain in VeroE6^TMPRSS2^ cells and HeLa^hACE2-TMPRSS2^ cells, using the procedures described previously^7,13,16,17^. Briefly, each of the purified IgG fraction was two-fold serially diluted in the culture medium. The diluted IgG fractions were incubated with 100 50% tissue culture infectious dose (TCID_50_) of the viruses at 37 °C for 20 min (final IgG dilution range: 6.3–100 µg/ml), after which the IgG-virus mixtures were inoculated into VeroE6^TMPRSS2^ cells and/or HeLa^hACE2-TMPRSS2^ cells (1.0 × 10^4^/well) in 96-well plates. The SARS-CoV-2 strains used in this assay were as follows: wild type strain, SARS-CoV-2^05-2N^ (PANGO lineage B), two alpha variants (SARS-CoV-2^QHN001^ and SARS-CoV-2^QK002^), beta variant SARS-CoV-2^TY8-612^, gamma variant SARS-CoV-2^TY7-501^, delta variant SARS-CoV-2^1734^, kappa variant SARS-CoV-2^5356^, omicron variant SARS-CoV-2^929-1N^ and R.1 variant SARS-CoV-2^76107^. After 3-day culture of the cells, the level of cytopathic effect (CPE) observed in SARS-CoV-2-exposed cells was determined using the WST-8 assay, employing Cell Counting Kit-8 (Dojindo, Kumamoto, Japan). The IgG antibody dilution that yielded 50% inhibition of CPE was defined as the 50% Inhibition Concentration (IC_50_). Each of the purified IgG fractions was tested in duplicate.

### Statistical analysis

Data are expressed as mean ± standard deviation (SD). Differences between groups were analyzed for statistical significance using Kruskal–Wallis test. When the latter test was significant, post-hoc Dunn’s multiple comparisons test was applied. Correlations between two assays were analyzed for statistical significance using nonparametric Spearman test. A *p* value < 0.05 denoted the presence of statistically significant difference. All statistical analyses were performed using the GraphPad Prism software version 8 (GraphPad Software, San Diego, CA).

### Institutional review board statement

The Ethics Committee at the NCGM approved the present study (#NCGM-G-003472-02). Each patient provided written informed consent. The study also conformed to the Declaration of Helsinki principles.

## Data Availability

The datasets generated during and/or analyzed during the study are available from the corresponding author on reasonable request.
